# In vitro measurements of radiation exposure with different modalities (computed tomography, cone beam computed tomography) for imaging the petrous bone with a pediatric anthropomorphic phantom

**DOI:** 10.1007/s00247-022-05308-8

**Published:** 2022-04-23

**Authors:** Beatrice Steiniger, Ursula Lechel, Jürgen R. Reichenbach, Martin Fiebich, Rene Aschenbach, Alexander Schegerer, Matthias Waginger, Anelyia Bobeva, Ulf Teichgräber, Hans-Joachim Mentzel

**Affiliations:** 1 Department of Diagnostic and Interventional Radiology, University Hospital, Am Klinikum 1, Jena, 07747 Germany; 2 MB3 External and Internal Dosimetry and Biokinetics, Federal Office for Radiation Protection, Neuherberg, Germany; 3 Medical Physics Group, Department of Diagnostic and Interventional Radiology, University Hospital, Jena, Germany; 4 Department LSE, Technische Hochschule Mittelhessen, Gießen, Germany; 5 Section Pediatric Radiology, Department of Diagnostic and Interventional Radiology, University Hospital, Jena, Germany

**Keywords:** Children, Cochlear implantation, Computed tomography, Cone beam computed tomography, Petrous bone, Radiation exposure

## Abstract

**Background:**

Various imaging modalities, such as multi-detector computed tomography (CT) and cone beam CT are commonly used in infants for the diagnosis of hearing loss and surgical planning of implantation hearing aid devices, with differing results.

**Objective:**

We compared three different imaging modalities available in our institution, including a high-class CT scanner, a mid-class CT scanner and an angiography system with a cone beam CT option, for image quality and radiation exposure in a phantom study.

**Materials and methods:**

While scanning an anthropomorphic phantom imitating a 1-year-old child with vendor-provided routine protocols, organ doses, surface doses and effective doses were determined for these three modalities with thermoluminescent dosimeters. The image quality was evaluated using the signal difference to noise ratio (SDNR) and the spatial resolution of a line-pair insert in the phantom head. The dose efficiency, defined as the ratio of SDNR and effective dose, was also compared.

**Results:**

The organ and surface doses were lowest with the high-class CT protocol, but the image quality was the worst. Image quality was best with the cone beam CT protocol, which, however, had the highest radiation exposure in this study, whereas the mid-class CT was in between.

**Conclusion:**

Based on our results, high-end CT should be used for surgical planning because it has the lowest dose, while the image quality is still sufficient for this purpose. However, if highest image quality is needed and required, e.g., by ENT surgeons, the other modalities should be considered.

## Introduction

The anatomy of the human lateral skull base is known to be complex. The most common middle/inner ear indications for lateral skull base imaging are suspected cholesteatoma, chronic otorrhea, evaluation of cochlear implant candidates, and electrode position control. Various radiation-based imaging modalities are used to assess bony structures [1, 2] and for surgical planning of hearing aid implantation in infants. Advances in multi-detector computed tomography (CT) technology allow acquisition of volumetric data with isotropic resolution and multi-planar reconstruction with resolutions greater than 16-line pairs per centimeter (LP/cm) [3]. This allows very detailed differentiation of the anatomical structures of the middle and inner ear (round window, oval window, ossicular chain, stapedial footplate, vestibular aqueduct, cochlea) [4]. Imaging of cochlear implants, however, is limited due to metal artifacts causing blurring around the electrodes on the images.

The preferred imaging technique should be selected based on answering the questions of how much information is needed, what structures need to be visualized, and how much noise can be tolerated. Ultra-high-resolution CT offers the best spatial resolution compared with conventional multi-detector CT due to smaller focus size and collimation, higher number of channels and detector rows, and larger matrix [5]. For children, age- and weight-adapted protocols with carefully selected size-based voltage and current settings are necessary [6]. Recently, conventional multi-detector CT has found strong competition from the use of cone-beam CT [6–9], which is widely used for isotropic high-resolution imaging and for endovascular interventional procedures [10]. Cone beam CT has emerged as an alternative for assessing bony structures of the lateral skull base as well as cochlear implants and stapes protheses, with potentially lower radiation dose than multi-detector CT. However, data on the feasibility, use and dose of cone beam CT in children are scarce [11].

The aim of this phantom study was to evaluate the dose efficiency for three different imaging modalities, including a high-end CT scanner, a mid-range CT scanner and an angiography system with cone beam CT option. Dose efficiency was defined as the ratio between the signal difference to noise ratio (SDNR) and the effective dose of a low-dose protocol for the high-class CT scanner, a standard routine protocol for the mid-class CT scanner, and a three-dimensional (3-D) cone beam CT protocol for the angiography system.

## Materials and methods

The modalities used in this phantom study included the multi-detector Revolution CT scanner (General Electric, Waukesha, WI) as the high-class CT, the Revolution Evo CT scanner (General Electric) as the mid-class CT and the flat panel CT Artis Q Zeego system (SIEMENS Healthineers, Erlangen, Germany) with cone beam CT option (DynaCT). The detector width is 256 lines (equivalent to 160 mm) for the Revolution CT and 64 lines (equivalent to 40 mm) for the Revolution Evo CT. The scan protocols were identical to those applied in our institution in daily routine for surgical planning or position monitoring in 2- to 3-year-old patients who are to receive or already have a cochlear implant. The acquisition parameters are listed in Table 1 and were compiled from the dose protocols, examination protocols and the digital imaging and communications in medicine (DICOM) headers of the image series. We used the routine protocols provided by the vendors for study purposes.

**Table 1:** These acquisition parameters were documented as dose protocols, examination protocols or in the digital imaging and communications in medicine (DICOM)-headers of the image series

	Revolution CT	Revolution Evo CT	DynaCT
tube current (mA)	80 fixed, ODM	75 fixed	216
tube voltage (kV)	100	100	109
additional filtration	small bowtie filter	small bowtie filter	0.0 mm Cu
scan range from (mm)	S38.579	S17.5	–
scan range to (mm)	I1.108	I18.5	–
scan length (mm)	39.7	36	39
SFOV (cm)	32	32	42
pitch	1	0.516	–
aquisition mode	sequenced	helical	–
noise index	21	21	–
pixel spacing (mm/mm)	0.342/0.342	0.391/0.391	0.380/0.380
slice thickness (mm)	0.625	0.625	0.38
filter kernel	Bone plus 2	Bone plus	HU\normal
views / rotation	2,496	984	496
iterative reconstruction level (%)	30	30	–
CTDI_vol_ (mGy)	7.9	15.7	–
DLP (mGy·cm)	31.6	110.7	–
DAP (cGy·cm^2^)	–	–	561.4

CTDI_vol_ volumetric computed tomography dose index, *DAP* dose area product, *DLP* dose length product, *HU* Hounsfield units, *ODM* organ dose modulation, *SFOV* scanned field of view

**Fig. 1:**
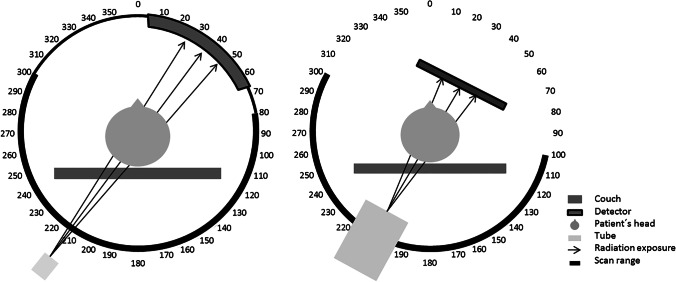
Schematic representation of the scan angles for the Revolution CT (left) and the DynaCT (right). The numbers around the circles represent the corresponding number of degrees. To reduce the dose to the eye lenses, the tube current is reduced to 70% of its original value in the angular scan range between 300° and 80° for the Revolution CT. For the DynaCT, the tube current is switched off in the frontal region between 300° and 100°

The parameters of the Revolution CT protocol are identical to those of a routine imaging protocol used daily in our institution. The size specifications of the volumetric CT dose index and the dose length product already consider over-beaming and over-ranging effects. For imaging with the DynaCT, a special 3-D head protocol specified by the manufacturer was used. For the acquisition, the glabellomeatal line was used as the reference plane to avoid lens exposure. The tube voltage and current are automatically adjusted to the anatomy of the phantom, only the field of view and the scan length are changeable by the operator. The Revolution CT scanner is equipped with organ dose modulation to reduce the dose to radiosensitive anterior organs (such as the eye lenses) by reducing the tube current to 70% when the X-ray tube is in anterior position in the angular 300° and 80° range (Fig. 1). The DynaCT protocol interrupts radiation exposure in the angular range between 300° and 100° of the scanned field (Fig. 1). Image reconstruction modes were chosen as implemented in the standard clinical protocols in use for each modality.

Dose measurements were performed with a pediatric anthropomorphic phantom (ATOM dosimetry phantom 704-D; CIRS, Norfolk, VA), containing homogenous tissue equivalent bone, lung and soft-tissue compositions for accurate X-ray attenuation characteristics. The head of the phantom consists of seven contiguous sections, each 2.5-cm thick and equipped with 5-mm diameter holes filled with tissue equivalent plugs for thermoluminescent dosimeter placement. The arrangement of the thermoluminescent dosimeters enables dosimetry in more than 20 internal organs. The phantom includes head, torso, arms and legs and represents a 1-year-old child (weight: 10 kg, height: 75 cm).

Absorbed doses were measured inside and on the surface of the phantom with lithium fluoride containing rods inside (1 ⋅ 1 ⋅ 6 mm^3^) and chips outside (3.2 ⋅ 3.2 ⋅ 0.9 mm^3^) (TLD100; Bicron-Harshaw, Solon, OH). The thermoluminescent dosimeters were calibrated using conventional X-ray equipment with tube voltages and filtration according to the tube settings of the angiography system or the multi-detector CT to approximate the radiation quality of the corresponding examinations. Individual calibration, annealing and readout of the thermoluminescent dosimeters were performed following a standard procedure using a Harshaw 5,500 TLD reader (ThermoFisher Scientific, Waltham, MA) and a PTW-TLDO oven (Physikalisch-technische Werkstätten Dr. Pychlau GmbH, Freiburg, Germany). The uncertainty for a single thermoluminescent dosimeter dose measurement was estimated to be 9% [12, 13].

**Fig. 2:**
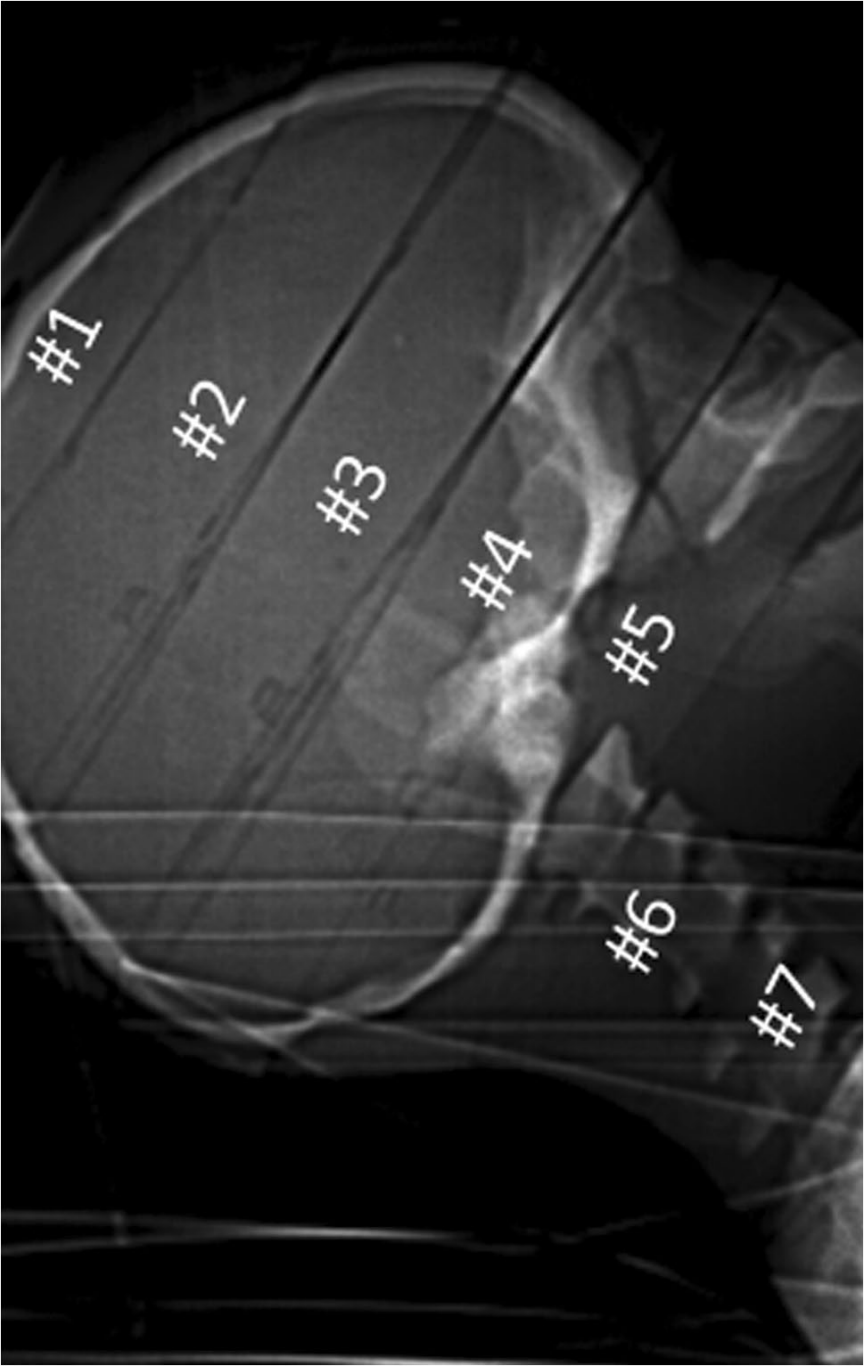
Phantom sections in a lateral topogram image. Due to the inclination of the head, different sections are exposed in the frontal and occipital regions

**Fig. 3:**
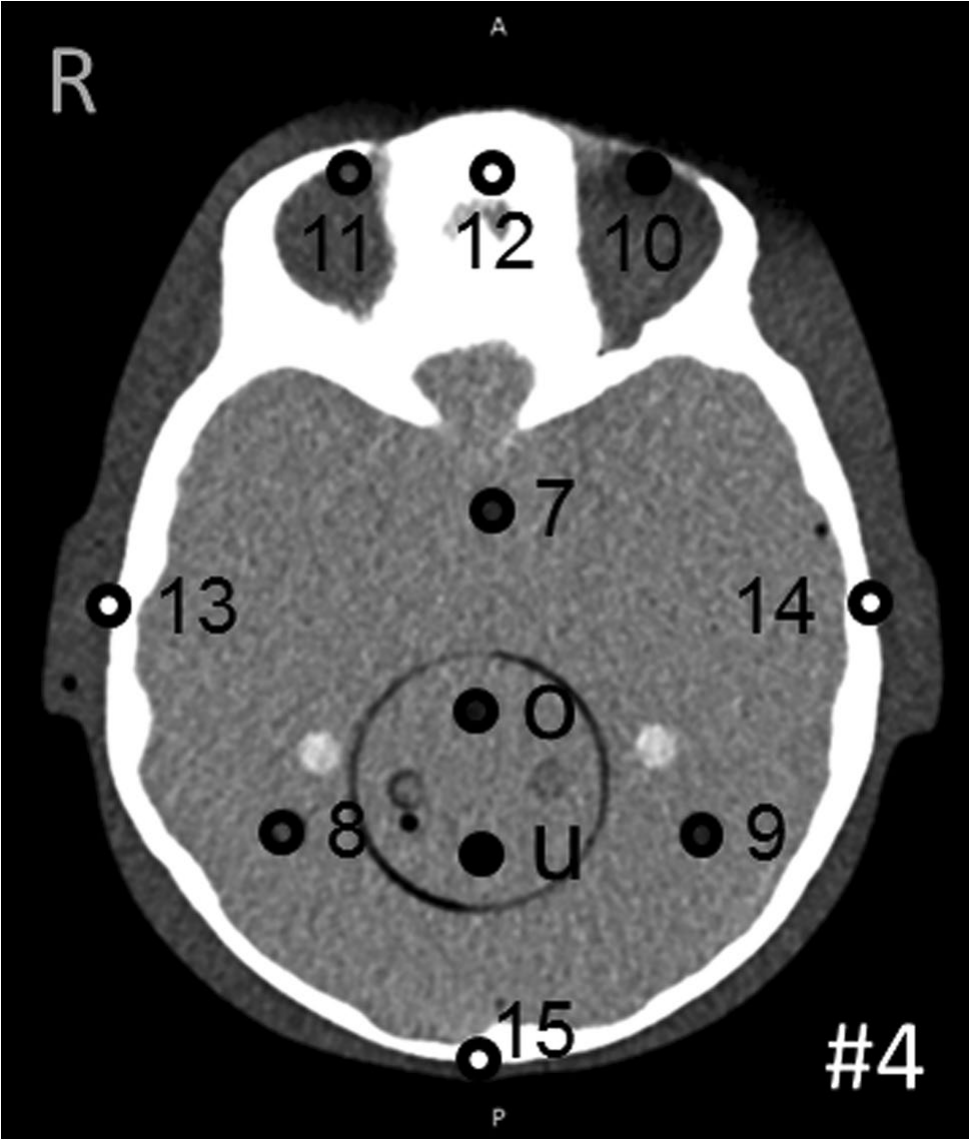
Example thermoluminescent dosimeter positions in section #4. The designation of each hole was specified by the manufacturer of the phantom. Correct assignment of each thermoluminescent dosimeter is important for precise determination of the organ doses

**Fig. 4:**
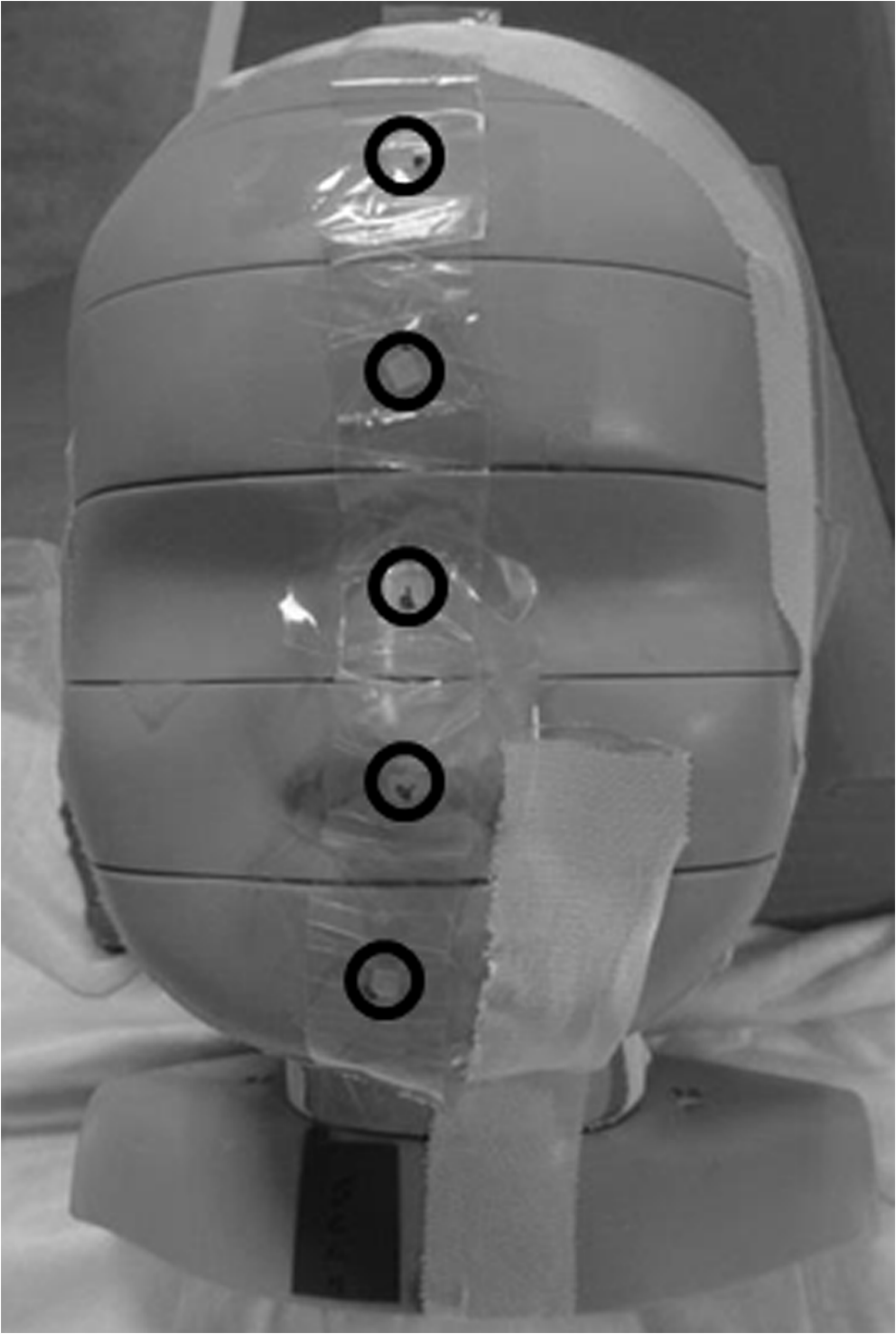
Placement of the surface thermoluminescent dosimeters (*black circles*) on the ventral surface (5 of the 7 thermoluminescent dosimeters are seen on the picture). Seven thermoluminescent dosimeters were placed analogously on the dorsal surface of the phantom

For each measurement, 24 thermoluminescent dosimeter rods were distributed in the head of the phantom (Figs. 2 and 3) and 14 thermoluminescent dosimeter chips on the surface of the head (7 ventral, 7 dorsal, one on each section, Fig. 4) to sample the non-uniform dose distribution. The designation of the individual holes was specified by the manufacturer of the phantom. Correct assignment of the thermoluminescent dosimeters is important for precise determination of the organ doses, which were calculated according to the recommendations of the manufacturer’s instructions for use of the phantom. The effective dose was calculated from the tissue and organ equivalent doses using the tissue weighting factors given in ICRP publication 103 [14].

Within the phantom sections #3 and #4, there is a cylindrical insert above both disks which contains optional holes for thermoluminescent dosimeter rods (o and u in Fig. 3) that can be replaced by a line pair (LP) target for quality assurance of CT imaging. A soft-tissue cylinder, containing five line pairs with spacing of 6, 8, 10, 11 and 12 LP/cm, was inserted to investigate spatial resolution. All images were scored by four qualified radiologists by assigning 1 point for “identifiable” and 0 points for “not identifiable” under evaluation conditions.

**Fig. 5:**
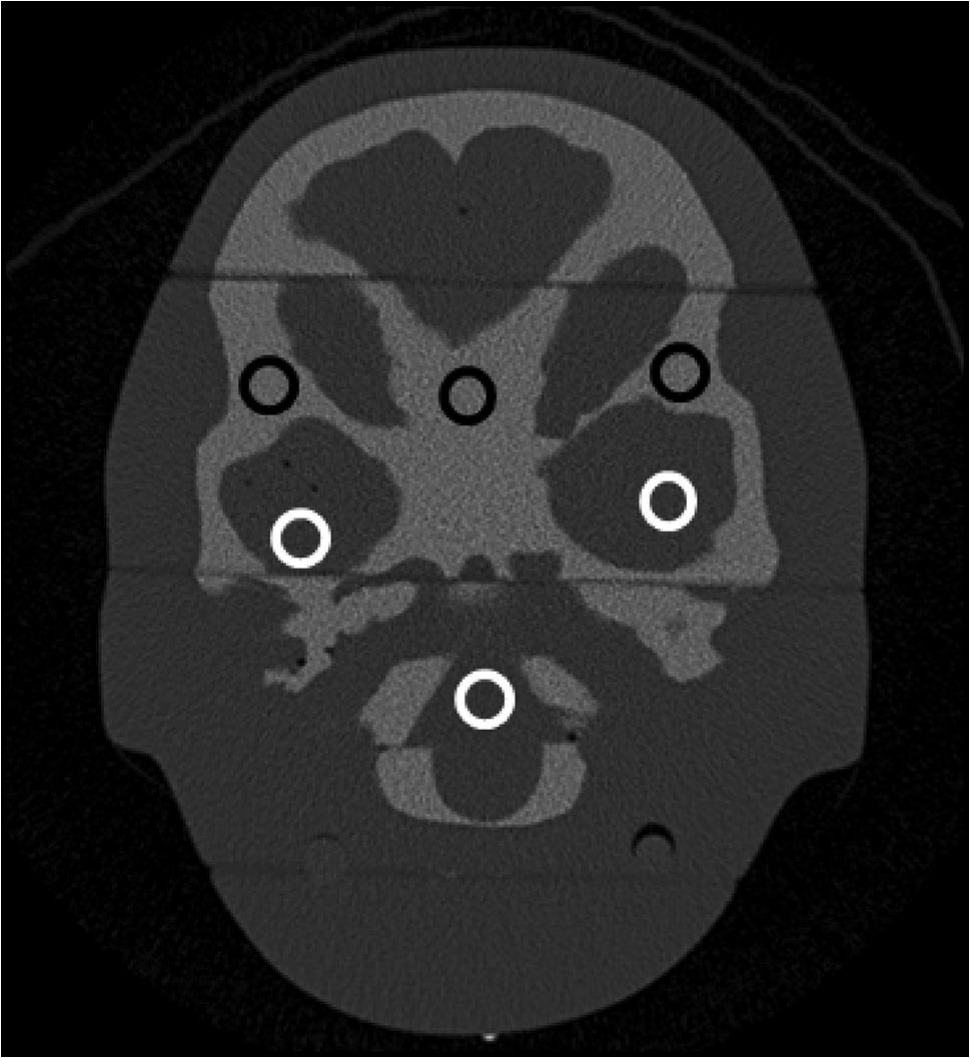
Example image (Revolution CT) with the regions of interest (*black circles* = bone, *white circles* = soft tissue) to determine the signal difference to noise ratio

Image quality was also quantitatively evaluated by determining the SDNR in a plane containing the petrous bone (Fig. 5). SDNR was determined by calculating the following ratio:

$$SDNR=\frac{\mid Mean\ HU\ of\ {ROI}_{bone}- Mean\ HU\ of\ {ROI}_{soft\ tissue}\mid }{\sqrt{\frac{\left( SD\ of\ {ROI}_{bone}\left)2+\right( SD\ of\kern0.5em {ROI}_{soft\ tissue}\right)2}{2}}}$$

Mean values (in Hounsfield units [HU]) and corresponding standard deviations (SD) were determined with the RadiAnt DICOM Viewer (Medixant, Poznan, Poland) from three circular regions of interest (ROIs) of bone and soft-tissue areas, all the same diameter (Fig. 5). For both tissues, the average values for the mean and standard deviations from the three corresponding regions of interest were used.

With the calculated SDNR, a simplified value for dose efficiency (DE) was estimated by taking the ratio between SDNR and the calculated effective dose for each examination:

$$DE=\frac{SDNR}{effective\ dose}$$

## Results

**Fig. 6:**
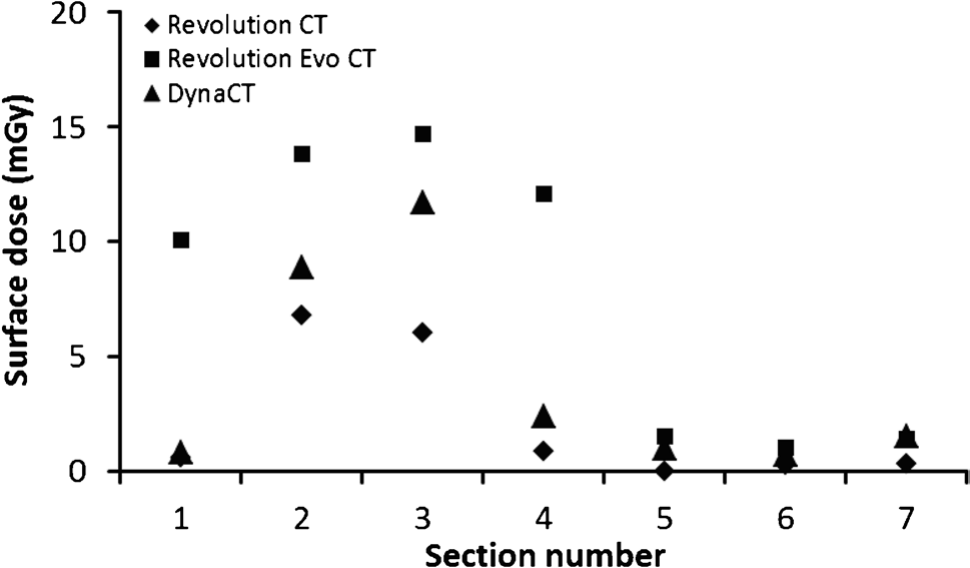
Surface doses for the frontal head region

**Fig. 7:**
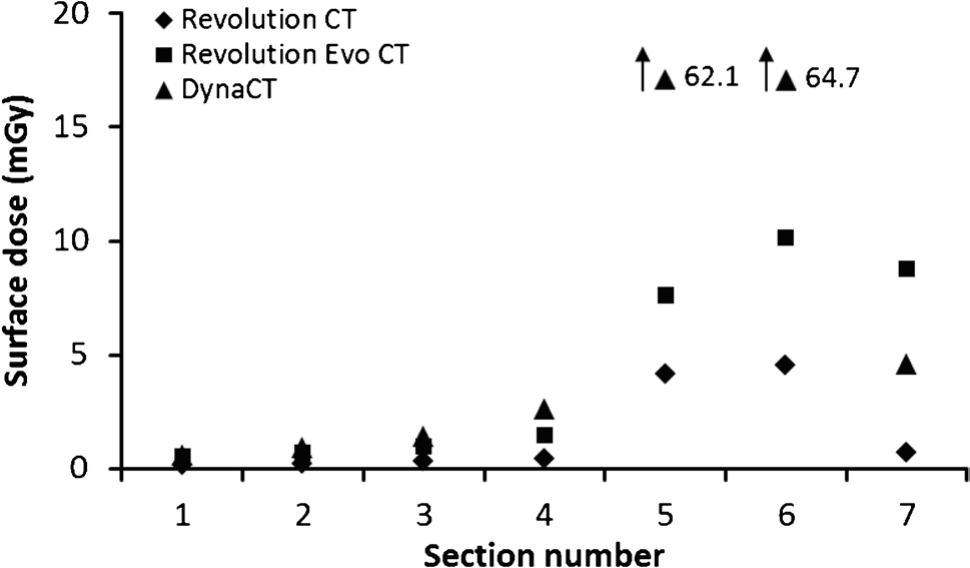
Surface doses for the occipital head region

As expected, radiation exposure in the scanned field of view was higher than in the peripheral regions but was different for the different modalities. For each thermoluminescent dosimeter position measured, the high-end CT yielded the lowest dose exposure. Exposure values for the Revolution Evo CT and the DynaCT were increased by more than 10-fold in some positions compared to the Revolution CT. Surface doses were also distinctly lower for the Revolution CT scan compared to the other two modalities (see Figs. 6 and 7).

**Fig. 8:**
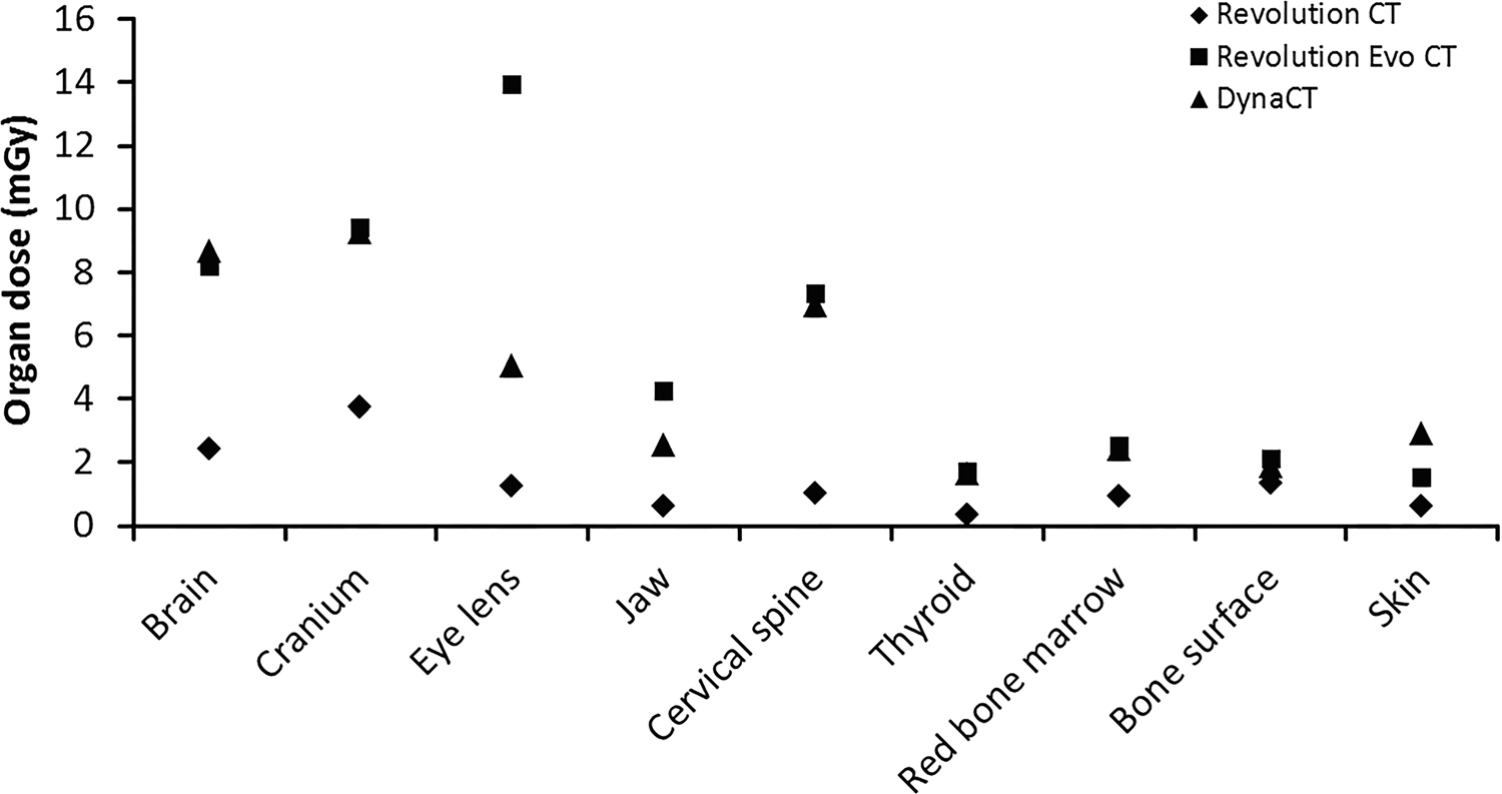
Organ doses for the different organs in the scan region for all modalities

Similar to the surface doses, organ doses were consistently lower with the Revolution CT scanner compared to the two other modalities (Fig. 8). This was most striking for brain, skull, eye lens, jaw and cervical spine. Particularly for the eye lens and the jaw, the organ doses differed substantially between the three modalities. The values for the other organ doses were more similar between the Revolution Evo CT and the DynaCT, although consistently higher compared to the Revolution CT scanner.

Table 2 shows the values for the HU and the associated SDs that were used to calculate the SDNR. The SD for the images on the Revolution CT is significantly larger than on the Revolution Evo CT and the DynaCT.

**Table 2:** Mean of Hounsfield units (HU) and root mean square deviation (SD) for the three region of interest measurements and the resulting signal difference to noise ratio (SDNR) determined with these values

	*mean ± root mean square deviation (n=3)*					
	Revolution CT		Revolution Evo CT		DynaCT	
HU bone	693.4	± 2.9	819.1	± 7.3	771.8	± 9.3
SD bone	278.1	± 7.0	92.8	± 9.5	25.0	± 2.4
HU soft tissue	39.3	± 6.2	44.2	± 8.0	86.5	± 5.3
SD soft tissue	185.3	± 5.5	73.5	± 2.6	21.4	± 0.6
SDNR	2.8		9.3		29.4	

*HU* Hounsfield units, *SD* root mean square deviation, *SDNR* signal difference to noise ratio

**Fig. 9:**
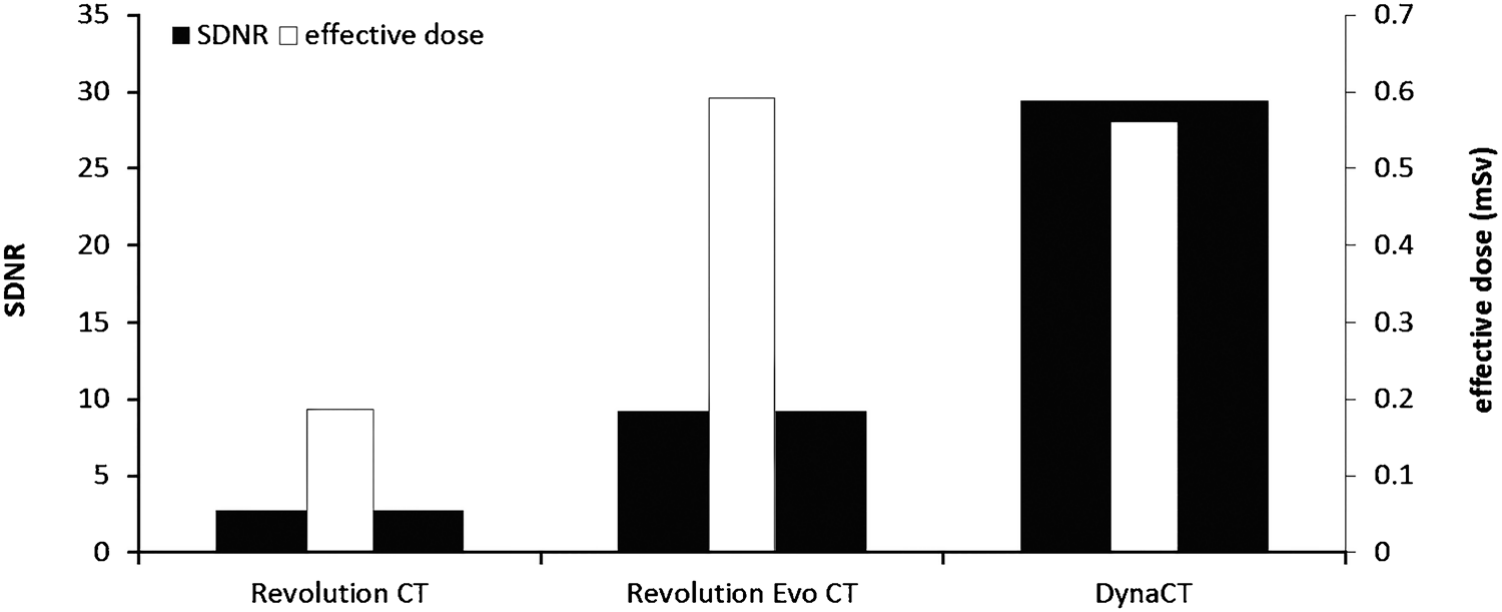
Calculated signal difference to noise ratio (SDNR) and effective doses for each modality

As Fig. 9 shows, both the SDNR (2.8) and the effective dose (0.2 mSv) were lowest for the Revolution CT. Although the SDNR is slightly higher for the Revolution Evo CT (9.3), the effective dose was the highest (0.6 mSv). The effective dose was similar (0.6 mSv) for the DynaCT, but image quality – as assessed by SDNR – was substantially improved (29.4).

The dose efficiency was comparable between the Revolution CT (15 mSv^−1^) and the Revolution Evo CT (16 mSv^−1^), but considerably higher for the DynaCT (52 mSv^−1^). The best ratio between image quality and radiation dose was achieved with the DynaCT.

**Fig. 10:**
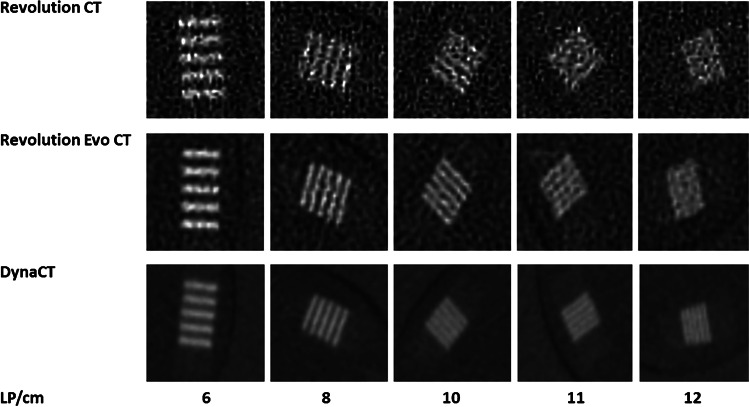
Spatial resolutions of the line pair inserts for the Revolution CT, Revolution Evo CT and the DynaCT, which were assessed by the radiologists. *LP* line pair

Like image quality as assessed by SDNR, the spatial resolution of the Revolution CT scan was distinctly worse than with the two other methods. Figure 10 shows the images of the different line pair inserts for the three scanners and Table 3 lists the corresponding visual scores in terms of line pair detectability in each scan series. The finest structure at 12 LP/cm was only identifiable with DynaCT.

**Table 3:** Total score of four radiologists, with one point awarded for five identifiable lines and no points for fewer than five identifiable lines

LP/cm	6	8	10	11	12
Revolution CT	4	4	4	4	0
Revolution Evo CT	4	4	4	4	1
DynaCT	4	4	4	4	4

LP/cm line pairs per centimeter

## Discussion

Dose optimization is an ever-present essential requirement in radiologic imaging, particularly in pediatric imaging applications. Because dose optimization and comparison studies in pediatric patients are not possible for ethical reasons, we used a pediatric-prepared head phantom in this study.

One previous phantom study reported effective doses of cone beam CT applications within a similar dose range as comparable to multi-detector CT protocols. With strong collimation on the pathology, a reduction of the cone beam CT dose is possible [15]. In regions with inherently high-contrast objects, like the temporal bone between air-filled spaces and bone, a reduced protocol with shorter rotation times and reduced image receptor dose can be applied [16]. Another effective way to reduce the applied dose is to reduce the rotation angle [17]. Cone beam CT with lower radiation in comparison to CT could therefore be of great advantage in children [18].

Looking at the spatial dose distribution in Fig. 6, the thermoluminescent dosimeters placed in the dorsal region of the phantom show the highest values with the DynaCT and, in relative terms, with the Revolution CT. In contrast, the dose values obtained with the Revolution Evo CT scan do not depend on a dorsal or ventral location. This observation is due to the applied scan protocol. The Revolution CT reduces the tube current to 70% of the “normal” dose level (without organ dose modulation) for gantry angles between 300° and 80° and the DynaCT even switches off exposure completely in the angle range between 300° and 100°. For scans with these two modalities, the three thermoluminescent dosimeter chips in the scanned field on phantom sections #5 to #7 measured the highest values. These high exposures in the dorsal region are necessary to preserve the organs in the frontal region of the head, especially the eyes and salivary glands. The fact that the largest dose load occurs in sections #1 to #4 in the frontal region is due to the forward tilted positioning of the phantom (Fig. 2) and is common when scanning patients.

The critical organ with petrous bone imaging is the eye lens. In this study, lens dose ranged from 1 to 14 mGy depending on the device. Bismuth shielding can be used to reduce the absorbed dose to the lens [19, 20]. The calculated organ doses for the lens demonstrate the imperative to have a scanning gap in the frontal region of the head. Considering image quality, this common radiation protection procedure should be used, if available. The protocol for the Revolution Evo CT scan can generally be adapted to the recommendations of the American Association of Physicists in Medicine for routine pediatric head CT to reduce dose while taking image quality into account [21]. The eye lens dose can also be further reduced by changing the positioning of the head [22]. For the specific conditions of petrous bone imaging, these protocols should be adapted. Because of the over-ranging aspect of multi-detector CT in helical mode, axial acquisition is preferred [23].

Regarding the two parameters used to assess image quality in this study, i.e. SDNR and spatial resolution, the Revolution CT protocol performed the worst. The DynaCT protocol performed the best while the Revolution Evo CT protocol was in between. Taking radiation exposure as the decisive measure, the decision whether high image quality is essential or whether medium (or even low) quality is sufficient must be made very carefully – and not only for pediatric patients. For example, although image quality was the worst with the Revolution CT protocol, it certainly is sufficient for surgical planning for inserting cochlear implants; for assessing correct placement, however, higher spatial resolution is necessary, such as that provided by the DynaCT protocol.

Nevertheless, when trading excellent image quality and high doses as seen with the cone beam CT protocol used in this study, it is essential to consider dose reductions while ideally maintaining the same image quality [24]. The dose exposure and efficiency values obtained in our study may vary with different cone beam CT scanners and optimized protocols. The scan length should be adjusted to the scan ranges of the other two modalities. For the two CT scanners, different filter kernels and higher reconstruction levels should be tested as first steps to optimize image quality. The pitch in the protocol of the Revolution Evo CT should be changed to a value of one. For all three modalities, the protocols need to be improved; however, to evaluate the proposed improvements and other conceivable optimizations, further testing is mandatory to ensure sufficient image quality.

A limitation of our study is that data collected on a phantom cannot be readily transferred to clinical practice. In addition to the technical conditions, the compliance of pediatric patients and thus the examination time and potential image degradation due to motion artefact are essential issues to be considered when evaluating the methods. For example, cone beam CT with its relatively long examination time is known to be very sensitive to patient movement, which is a weakness of this modality and may limit its applicability in real-life situations.

## Conclusion

Based on the evaluation of scan protocols for three imaging modalities regarding radiation exposure and image quality for petrous bone imaging, we recommend the use of the Revolution CT in our institution for the planning of hearing aid device implantations. For postoperative position control or when higher spatial resolution and/or lower noise are required, the other two modalities should be considered. The DynaCT protocol in the tested configuration does not seem suitable for pediatric patients due to the substantial radiation exposure and should -- if at all -- only be used in exceptional cases. The parameters of all tested scan protocols must be optimized with respect to image quality and radiation exposure.
